# Validation of skinfold equations and alternative methods for the determination of fat-free mass in young athletes

**DOI:** 10.3389/fspor.2023.1240252

**Published:** 2023-08-11

**Authors:** Andrew R. Jagim, Grant M. Tinsley, Brandon R. Merfeld, Abby Ambrosius, Chinguun Khurelbaatar, Christopher Dodge, Makenna Carpenter, Joel Luedke, Jacob L. Erickson, Jennifer B. Fields, Margaret T. Jones

**Affiliations:** ^1^ Sports Medicine, Mayo Clinic Health System, Onalaska, WI, United States; ^2^ Exercise & Sport Science, University of Wisconsin—La Crosse, La Crosse, WI, United States; ^3^ Energy Balance & Body Composition Laboratory, Department of Kinesiology & Sport Management, Texas Tech University, Lubbock, TX, United States; ^4^ Department of Exercise Science and Athletic Training, Springfield College, Springfield, MA, United States; ^5^ Patriot Performance Laboratory, Frank Pettrone Center for Sports Performance, George Mason University, Fairfax, VA, United States; ^6^ Sport, Recreation, and Tourism Management, George Mason University, Fairfax, VA, United States

**Keywords:** body composition, skinfold, fat-free mass, youth athlete, minimum wrestling weight

## Abstract

**Intoduction:**

To cross-validate skinfold (SKF) equations, impedance devices, and air-displacement plethysmography (ADP) for the determination of fat-free mass (FFM).

**Methods:**

Male and female youth athletes were evaluated (*n* = 91[mean ± SD] age: 18.19 ± 2.37 year; height: 172.1 ± 9.8 cm; body mass: 68.9 ± 14.5 kg; BMI: 23.15 ± 3.2 kg m^−2^; body fat: 19.59 ± 6.9%) using underwater weighing (UWW), ADP, and SKF assessments. A 3-compartment (3C) model (i.e., UWW and total body water) served as the criterion, and alternate body density (Db) estimates from ADP and multiple SKF equations were obtained. Validity metrics were examined to establish each method's performance. Bioelectrical impedance analysis (BIA), bioimpedance spectroscopy (BIS), and the SKF equations of Devrim-Lanpir, Durnin and Womersley, Jackson and Pollock (7-site), Katch, Loftin, Lohman, Slaughter, and Thorland differed from criterion.

**Results:**

For females, Pearson's correlations between the 3C model and alternate methods ranged from 0.51 to 0.92, the Lin's concordance correlation coefficient (CCC) ranged from 0.41 to 0.89, with standard error of the estimate (SEE) ranges of 1.9–4.6 kg. For SKF, the Evans 7-site and J&P 3 Site equations performed best with CCC and SEE values of 0.82, 2.01 kg and 0.78, 2.21 kg, respectively. For males, Pearson's correlations between the 3C model and alternate methods ranged from 0.50 to 0.95, CCC ranges of 0.46–0.94, and SEE ranges of 3.3–7.6 kg. For SKF, the Evans 3-site equation performed best with a mean difference of 1.8 (3.56) kg and a CCC of 0.93.

**Discussion:**

The Evans 7-site and 3-site SKF equations performed best for female and male athletes, respectively. The field 3C model can provide an alternative measure of FFM when necessary.

## 1. Introduction

Body composition measurement is an important assessment technique in sports. Body composition parameters can provide valuable information regarding the amount and ratios of certain tissue components within the body, depending on the instrumentation used. In general, parameters such as body fat percentage, fat-free mass, and lean body mass are measured directly, or estimated based on prediction equations or indirect measurements. In athletic settings, laboratory-grade equipment is often not available or a feasible option due to cost, technician requirements, and time, particularly in high school or collegiate settings. Therefore, several field-based methodologies have been used to provide estimates of body composition parameters. Skinfold measurements and bioimpedance devices are examples of field-based assessment techniques that have been cross-validated against criterion measures in youth athletes and young adult populations with mixed findings (1–7). Skinfold measurement utilizes a caliper to obtain a measure of subcutaneous adiposity (i.e., skinfold thickness) at select sites throughout the body, using a double fold of gripped skin. These measures are then summed and used in prediction equations to estimate body density and subsequently fat mass (7). Bioimpedance methods can be classified as single (bioelectrical impedance analysis) vs. multi-frequency (bioimpedance spectroscopy) devices, and rely on the use of electrical current transmission through the body to calculate impedance and reactance of the current (6). BIA devices then utilize regression equations to estimate specific body composition compartments, whereas BIS devices use Cole modeling (8) and mixture theories (9) to estimate body water and other body compartments, of which the latter is often regarded as the more accurate of the two for evaluating body composition parameters (6).

Body weight and composition assessment can play a pivotal role in certain sports (e.g., weight-category sports and combat sports). For example, during the pre-season, wrestlers are required to complete a body composition assessment (most commonly via skinfold measures) to estimate body fat percentage, which is then used to calculate minimal wrestling weight (MWW) as part of a weight certification program. At the time of assessment, a wrestler's weight and body fat percentage are used to extrapolate what their weight would be (assuming no changes in fat-free mass), if they were to wrestle at a body fat percentage of 7% (5% at the collegiate level) and 12% for males and females, respectively, for determination of their MWW. Despite recommendations for safe and conservative weight loss strategies, research shows that wrestlers and weight-class athletes still rely on unhealthy practices including extended fasting periods, dehydration strategies, excessive exercise, and weight loss pills or laxatives (10–12), which can pose a significant risk to athlete health and well-being. Therefore, the establishment of accurate body composition assessment strategies to determine safe and appropriate weight class decisions for wrestlers is imperative.

The accuracy of skinfold prediction equations and other body composition methodologies for the measurement of body fat percentage has previously been evaluated (13–17); however, concerns have been raised in regard to the accuracy of this process (18), as there are potential sources for error. In wrestling, this could result in athletes being allowed to compete in too low of a weight class. Concerns include the accuracy of the prediction equations used to estimate body density, testing error, and the potential for variability when using skinfold measures for body fat percentage determination (19–21). For example, previous research in children reported significant sources of error and directional bias when utilizing skinfold thickness to estimate body fat percent (22). Similar conclusions have been reported in highly trained young adult populations, in that skinfold-derived measures of body fat percent do not yield accurate measures of fat mass and fat-free mass; particularly when used to track changes in body composition parameters (23). Furthermore, several of the skinfold equations may not be valid for young female athletes.

In an effort to improve the weight certification program, there have been investigations into the accuracy of skinfold prediction equations along with other minimally invasive and cost-efficient modes of body composition assessment (16, 24–26). At the collegiate level, the examination of various modes of body composition assessment for MWW determination have yielded differences in minimum weights up to 3.3 kg, which may result in inappropriately categorized wrestlers. Cutrufello et al. (25) recently found standard error of estimate values ranging from 2.4 to 3.2 kg when determining MWW, depending upon the technique used. Similar discrepancies have been reported when comparing body fat percent values derived from 12 different skinfold prediction equations among Olympic wrestlers (26), indicating the selection of body composition technique, or equations, might influence the resulting MWW values.

Advancements in body composition assessment techniques may improve the accuracy of field-based measures. By cross-validating prior methods using a 3-compartment model as a criterion measure, while also exploring novel techniques, improvements to the weight certification program may be possible. As a result, there may be opportunities to refine the current process for MWW determination within the sport of wrestling. Therefore, the purpose of the current study was threefold: (A) to determine the most accurate skinfold prediction equations for young male and female athletes, respectively, using a three-compartment model of body composition assessment; (B) to examine the utility of alternative modes of body composition assessment compared to criterion measures; and (C) to identify the number of athletes that may be mis-categorized to a certain weight-class when current methods are compared to criterion measures.

## 2. Methods

### 2.1. Study design

Subjects completed a battery of body composition assessments during a single morning of testing, including skinfold (SKF), underwater weighing (UWW), air displacement plethysmography (ADP), bioimpedance spectroscopy (BIS), multi-frequency BIA (MFBIA), and two single-frequency BIA (SFBIA) analyzers. Prior to testing, subjects were asked to refrain from intense activity (>24 h.) and food (>8 h.). Upon arrival, subjects provided a urine sample to determine adequate hydration status (USG < 1.02). Athletes with a urine specific gravity (USG) > 1.02 the morning of testing were re-scheduled. Three-component model estimates of %BF (%BF3C) included total body water from BIS measurement, and body density by UWW. Skinfold (SKF) measures at 8 sites were completed in triplicate. Fat-free mass (FFM) was assessed via each mode of body composition analysis and used to calculate minimum wrestling weight based on a 7.0% minimum body fat for males and 12.0% body fat for females. A body fat percentage value was also assessed using a field-based 3-compartment model of body composition, derived from the SKF and BIA results. Cross-validation occurred for all modes of body composition assessment, using the 3C measures of FFM and MWW as criterion measures. Target outcomes included FFM and MWW.

### 2.2. Subjects

Ninety-one male and female youth athletes (age range: 14–24 years of age) were evaluated (female, *n* = 51; [mean ± SD] age: 18.19 ± 2.37 year; height: 172.1 ± 9.8 cm; body mass: 68.9 ± 14.5 kg; BMI: 23.15 ± 3.2 kg/m^2^; body fat: 19.59 ± 6.9%) participated. Because of the novelty of female wrestling and subsequent limited sample size available, athletes of all sport types were recruited from local high schools and Universities in the southwest region of Wisconsin, USA. All interested participants and their parents or guardians were invited to attend an informational meeting at which time, the details of the study, participant involvement, benefits, risks, and projected outcomes were discussed. Participants who were actively cleared to participate in a high school (*n* = 41) or collegiate (*n* = 50) sports were included in the study. Further inclusion criteria included being between the ages of 14–25 years of age. Exclusion criteria included pregnancy or breastfeeding, and currently being treated for or diagnosed with a cardiac, respiratory, circulatory, autoimmune, musculoskeletal, metabolic, hematological, neurological, or endocrine disorder or disease. Athletes from baseball (*n* = 7), wrestling (*n* = 9), football (*n* = 12), basketball (*n* = 4), soccer (*n* = 28), track/cross-country (*n* = 10), weight/power lifting (*n* = 4), volleyball (*n* = 5), gymnastics (*n* = 5), dance (*n* = 1), softball (*n* = 2), tennis (*n* = 1), CrossFit (*n* = 1), skiing (*n* = 1), and hockey (*n* = 1) were represented. 40 (43.9%) athletes participated in two sports, 19 (20.8%) in three sports, and 3 (3.3%) in four sports. The study was conducted according to the Declaration of Helsinki guidelines, and procedures were approved by the University's Institutional Review Board for use of human subjects in research. All participants signed an informed consent or assent (for those <18 years of age) document prior to participation. Parental/guardian consent was provided for participants <18 years of age.

### 2.3. Study procedures

#### 2.3.1. Anthropometrics, skinfold and body density

Body mass and height were initially assessed using a self-calibrating physician's scale and stadiometer to the nearest 0.1 kg and 0.5 cm, respectively. Skinfold measures were conducted three times (to the nearest 0.1 mm) using a Harpenden Skinfold caliper across an 8-site model (subscapular, triceps, chest, midaxillary, suprailiac, abdominal, thigh and biceps). Skinfold technician test-retest reliability in the current study was ICC: 0.991 (95% CI: 0.987, 0.994).

#### 2.3.2. Under water weighing

Residual volume was determined in the UWW tank with subjects immersed at shoulder level using a closed-circuit oxygen dilution method (27). Prior to each test, the system was calibrated, and the rebreathing bag was flushed out with oxygen and emptied with a vacuum pump. An electronic nitrogen analyzer (*Med Science 505 Nitralyzer, Needham Heights, MA*) was used to measure gas exchange while the subject was inspiring and expiring through the bag for multiple cycles. Next, the subject was instructed to place a nose clip on and to seal their lips tightly around the mouthpiece and breathe normally. The subject was then instructed to forcefully expire as much air as possible. When the subject expired all their air, they signaled the technician, and then a valve was opened, which connected the subject to the rebreathing bag. Once connected, the subject was instructed to deeply breathe in, followed by deep, rapid breaths in and out until an equilibrium was displayed on the electronic dashboard. The residual volume was then calculated using the following equation from Wilmore 1969 (27):

$$\mathrm{Residual}\;\mathrm{Volume}=(VO_{2})(\mathrm{EN}-\mathrm{IN})/[\mathrm{AN}-\mathrm{FN}-\mathrm{RVDS}]$$

VO_2_ = Initial volume of O2 in spirometer system including dead space between breathing valve and spirometerbell (.034 L); EN = Percent nitrogen at equilibrium; IN = Impurity of nitrogen; AN = Percent of alveolar nitrogen; FN = Percent of final nitrogen; RVDS = mouthpiece dead space (.070 L).

Electronic load cells suspended an underwater chair to assess the subject's weight underwater. An automated computer program converted the voltage measured at the load cell into weight in kilograms. The computer used an average of 100 readings per trial to determine a value that represented the subject's weight while submerged in the water. The UWW weighing chair was calibrated prior to each test. Following determination of residual volume, the subject stepped off the chair placed their back against the side of the tank with the water level at the neck. With the subject off the chair, and motionless, the computer zeroed the UWW chair. Next, two 2 kg weights were placed on each side of the chair while the system calibrated the load cells to 4 kg. After calibration, the weights were removed, and the subject assumed the position in the UWW chair. The subject was then instructed to exhale as much air as possible, while slowly submerging until their head was totally submerged (5–10 cm below water level). Once air bubbles stopped appearing, the computer recorded the weight and the technician tapped on the side of the tank, signaling to the subject to come up for air. This procedure was repeated 5–10 times in order for the subject to produce a consistent UWW with an average of 2–4 trials (within 0.5 kg) calculated for the final UWW.

#### 2.3.3. Air displacement plethysmography

Body composition variables (i.e., %BF, fat-free mass, fat mass and body density) were assessed using air displacement plethysmography (*BOD POD model 2000A; BOD POD; Cosmed USA, Concord, CA*) according to standard operating procedures. Athletes were instructed to wear spandex or form-fitting clothing and wore a lycra swim cap. All jewelry was removed prior to testing. Thoracic gas volume was predicted using manufacture settings. Previous test-to-test reliability results for the use of ADP assessment in athletes has yielded high reliability for BM (*r* = 0.999), body fat percent (*r* = 0.994), and FFM (*r* = 0.998) in our laboratory.

#### 2.3.4. Bioelectrical impedance and total body water

Whole body SFBIA measurements were assessed using a 50 kHz device (*Quantum IV, RJL systems, Clinton MI)* to determine resistance (R), which was used to estimate body composition through select validated equations as later described. Total body water (TBW), extracellular water (ECW) and intracellular water (ICW) were assessed using BIS (*SFB7, ImpediMed, Carlsbad, CA*) with 256 measurement frequencies to model the fluid content of the body by obtaining total body water estimates. BIS utilizes Cole modeling (8) and mixture theories (9) to predict body fluids rather than regression equations used by BIA techniques. Coefficients utilized for males (*ρe* = 273.9, *ρi* = 937.2) and females (*ρe* = 235.5, *ρi* = 894.2), as well as body density, body proportion and hydration values (1.05, 4.30 and 0.732, respectively) were the same as those utilized in previous investigations with the selected BIS analyzer (28, 29). These SFBIA and BIS measurements were taken with the participant in the supine position prior to assessment using manufacturer-recommended hand-to-foot electrode arrangement. Alcohol wipes were used prior to placement of the adhesive electrodes. Previous research has indicated that TBW measures derived from similar BIS units have yielded strong agreement (*r* = 0.90; SEE = 2.65l; TE = 2.6l) compared with deuterium dilution criterion measures (30), with test-to-retest reliability producing a SEM of 0.48 L and an ICC of 0.99 (31). Body composition was also assessed using a consumer-grade MFBIA device, the H20N scale (*InBody Inc., Cerritos, CA*) and a foot-to-foot SFBIA device [(F2FBIA) *Tanita BF-679W, IL, USA*]. Subjects completed two measurements on each device with an average of the two used for analysis.

#### 2.3.5. Body density estimation

Body density (*D_b_*) values expressed in kg/L were obtained from UWW, ADP, and multiple SKF equations (Table 1).

**Table 1:** Summary of equations to predict body density or body fat percent.

Author	Equation
Jackson and Pollock 3-site and 7-site (32, 33)	3-site $\mathrm{Males}:D_{b}=1.10938-(0.0008267\times \mathrm{SS}3)+(0.0000016\times \mathrm{SS}3^{2})-(0.0002574\times \mathrm{age})$ $\mathrm{Females}:D_{b}=1.0994921-(0.0009929\times \mathrm{SS}3)\;+\;(0.0000023\times \mathrm{SS}3^{2})-(0.0001392\times \mathrm{age})$ Where SS3 is, for males, the sum of chest, abdomen, and thigh skinfolds; and, for females, the sum of triceps, thigh, and suprailium skinfolds. 7-site $\mathrm{Males}:D_{b}=1.112-(0.00043499\times \mathrm{SS}7)+(0.00000055\times \mathrm{SS}7^{2})-(0.00028826\times \mathrm{age})$ $\mathrm{Females}:D_{b}=1.0970-(0.00046971\times \mathrm{SS}7)+(0.00000056\times \mathrm{SS}7^{2})-(0.00012828\times \mathrm{age})$ Where SS7 is the sum of seven skinfolds (chest, midaxillary, triceps, subscapular, abdomen, suprailium, and thigh).
Forsyth (34)	$D_{b}=1.10647-(0.00162\times \mathrm{Subscapular})-(0.00144\times \mathrm{Abdomen})-(0.00077\times \mathrm{Triceps})+(0.00071\times \mathrm{Midaxillary})$
Katch (35)	$D_{b}=1.09665-(0.00103\times \mathrm{Triceps})-(0.00056\times \mathrm{Subscapular})-(0.00054\times \mathrm{Abdomen})$
Lohman (36)	$D_{b}=1.0982-(0.000815\times (\mathrm{Triceps}+\mathrm{Subscapular}+\mathrm{abdomen}\_\mathrm{av}))+(0.00000084\times (\mathrm{Triceps}+\mathrm{Subscapular}\;+\mathrm{Abdomen}{)}^{2})$
Thorland (37)	$D_{b}=1.1136-\;(0.00154\times (\mathrm{Triceps}\times \mathrm{Subscapular}\times \mathrm{Midaxillary}))+(0.00000516\times (\mathrm{Triceps}+\mathrm{Subscapular}\;+\mathrm{Midaxillary}{)}^{2})$
Durnin and Womersle (38)	Multiple equations were developed by Durnin and Womersley, with the general form of: $D_{b}=c-(m*\log(\mathrm{skinfold}))$ Accordingly, the equations for males up to 19 years and between 20 and 29 years were: $D_{b}=1.1620-(0.0630\times \log(\mathrm{SS}4))$ $D_{b}=1.1631-(0.0632\times \log(\mathrm{SS}4))$ The equations for females up to 19 years and between 20 and 29 years were: $D_{b}=1.1549-(0.0678\times \log(\mathrm{SS}4))$ $D_{b}=1.1599-(0.0717\times \log(\mathrm{SS}4))$
	Bioimpedance and anthropometric equations to estimate BF%
Matias (39)	$\mathrm{FFM}=-2.261+\left(0.327\times \frac{\mathrm{Heigh}t^{2}}{R}\right)+(0.525\times \mathrm{BM})+5.462\times \mathrm{Sex}$ Where height is expressed in centimeters, *R* is resistance expressed in ohms, BM is expressed in kilograms, and sex = 0 for females and sex = 1 for males.
Stewart (males only) (40)	$\mathrm{FFM}=\frac{294.3\times \mathrm{Heigh}t^{2}}{R}+(662.7\times \mathrm{BM})+(71.8\times \mathrm{Xc})+662.7$ Where height is expressed in meters, *R* is resistance in ohms, Xc is reactance in ohms, and BM is expressed in kilograms.
Fornetti et al. (females only) (41)	FFM=(0.143×Height)+(0.565×BM)−10.03, where height is expressed in cm and BM is expressed in kg.
Loftin et al. (females only) (42) Slaughter (females only) (43)	BF%=−23.39+(2.27×BMI)+(1.94×triceps)−(2.95×Race)−(0.52×age)−(0.06×BMI×triceps) Where race = 1 if Black/African American and race = 0 for other races. If sum of skinfolds ≤35 mm BF%=(0.546×(Triceps+Subscapular))+9.7 If sum of skinfolds >35 mm $\mathrm{BF}\text{\%}=(1.33\times (\mathrm{Triceps}+\mathrm{Subscapular}))-(0.013\times {\left(\mathrm{Triceps}+\mathrm{Subscapular}\right)}^{2})-2.4$
Evans 3-site and 7-site (1)	BF%=8.997+(0.24658×SS3)−(6.343×Sex)−(1.998×Race) BF%=10.566+(0.12077×SS7)−(8.057×Sex)−(2.545×Race) Where sex = 0 for females and sex = 1 for males; race = 0 for Caucasian and race = 1 for Black; SS3 is the sum of abdomen, thigh, and triceps skinfolds; and SS7 is the sum of seven skinfolds.
Devrim-Lanpir (29)	BF%=0.30+(0.72×abdomen)+(11.43×sex) Where sex = 0 for males and sex = 1 for females.

*D_b_*, body density; *c* and *m* are values specific to each skinfold or sum of skinfolds, as well as age and sex. For the present analysis, the equations using the sum of four skinfolds (SS4; biceps, triceps, subscapular, and suprailium) were used. For males, the age ranges of Durnin and Womersley used in the present analysis were 17–19 years—for all male participants up to 19 years—and 20–29 years for participants in this range. For females, the age ranges of Durnin and Womersley used in the present analysis were 16–19 years—for all female participants up to 19 years—and 20–29 years for participants in this age range. FFM, fat-free mass; BM, body mass; R, reactance; BF%, body fat percent.

#### 2.3.6. Body composition estimation

Data from UWW, ADP, and SKF were used in several body composition estimation equations. For all equations producing a BF% value, the corresponding FFM value was then calculated manually as follows:

$$\mathrm{FFM}=\mathrm{BM}-\left(\mathrm{BM}\times \frac{\mathrm{BF}\text{\%}}{100}\right)$$

For UWW and ADP, BF% values were produced using the Siri (44) and Brozek (45) equations, respectively:

$$\mathrm{BF}\text{\%}=\left[\left(\frac{4.95}{D_{b}}\right)-4.5\right]\times 100$$

$$BF\text{\%}=\left[\left(\frac{4.57}{D_{b}}\right)-4.142\right]\times 100$$

For the remaining SKF *D_b_* equations, BF% and subsequent FFM estimates were obtained using the Siri 2C equation only.

Body composition estimates from F2FBIA (Tanita), MFBIA (InBody), and BIS (ImpediMed) were used, along with additional estimates obtained using SFBIA (RJL) raw bioimpedance in two FFM prediction equations (Table 1). Several equations predicting BF% utilizing anthropometric parameters were also employed (Table 1).

#### 2.3.7. Criterion method

For the criterion 3C model, *D_b_* was taken from UWW, TBW was taken from BIS, and BM was taken from the calibrated scale. A field-based 3C model, *D_b_* was taken from SKF, TBW was estimated from bioelectrical resistance from SFBIA (RJL), and BM was taken from the calibrated scale. The TBW estimate from SFBIA (RJL) was obtained using the Matias et al. equation (46):

$$\mathrm{TBW}=0.286+\left(0.195\times \frac{\mathrm{Heigh}t^{2}}{R}\right)+(0.385\times \mathrm{BM})+(5.086\times \mathrm{Sex})$$

Where sex = 0 for females and sex = 1 for males.

Two estimates were produced using the Siri 3-compartment model equation (47):

$$\mathrm{BF}\text{\%}=100\times \left(\left(\frac{2.118}{D_{b}}\right)-\left(0.78\times \frac{\mathrm{TBW}}{\mathrm{BM}}\right)-1.354\right)$$

This 3-compartment model has been previously used in college-aged men and women, with a total error of measurement value of 0.1152%fat and 0.1152%fat, respectively (48, 49).

#### 2.3.8. Minimal wrestling weight

For all methods, BF% estimates were used to calculate minimum wrestling weight (MWW) based on the minimum requirement of 7% body fat for males and 12% body fat for females. MWW was estimated as:

$$\mathrm{Males}\;\mathrm{MWW}=\frac{\left(1-\frac{\mathrm{BF}\text{\%}}{100}\right)\times \mathrm{BM}}{0.93}$$

$$\mathrm{Females}\;\mathrm{MWW}=\frac{\left(1-\frac{\mathrm{BF}\text{\%}}{100}\right)\times \mathrm{BM}}{0.88}.$$

## 3. Statistical analysis

Separate analyses were performed for males and females. The same statistical analysis procedures were performed for fat-free mass and minimal wrestling weight estimates. To determine which methods differed from the criterion method (3C model with UWW *D_b_* and BIS TBW), a one-way analysis of variance (ANOVA) test with repeated measures was performed, with the body composition assessment method specified as a within-subjects factor. Significant effects were followed up with pairwise *t*-tests, with the criterion 3C model specified as the reference group and using the Holm adjustment for multiple comparisons. This analysis was performed using the *rstatix* package for R (v. 4.1.2) (50). Additionally, equivalence testing was performed to determine which methods were statistically equivalent to the criterion method. The *TOSTER* (51) R package was used for this analysis, and equivalence intervals were set at ±2 kg. The entire 90% two one-sided test (TOST) confidence interval was required to fall within the specified equivalence interval for equivalence to be demonstrated.

Bland–Altman analysis (52) with linear regression was performed to identify proportional bias, and the 95% limits of agreement were calculated to indicate individual-level error. The mean difference between the criterion and alternate methods was also calculated. Correlations between the criterion method and alternate methods were established using Pearson's *r* and Lin's concordance correlation coefficient (CCC) (53, 54). The standard error of the estimate (SEE) was estimated via regression procedures. An *a priori* power analysis determined that a sample size of 46 subjects per group (male and female) would be needed for a Type I error at *α* = 0.05 and a power of 80%, for an expected Pearson correlation coefficient of *r* = 0.60 based on previous studies evaluating the reliability of body composition assessment methods (25, 55). All analyses were performed in R (v. 4.1.2), and statistical significance was accepted at *p *< 0.05.

## 4. Results

### 4.1. Fat-free mass

#### 4.1.1. Females

FFM estimates are displayed in Table 2.

**Table 2:** Fat-free mass estimates for male and female athletes.

Method	Females (*n* = 51)				Males (*n* = 40)			
	Mean (kg)	SD	MD (kg)	SD of MD	Mean (kg)	SD	MD (kg)	SD of MD
3C	47.9	4.4	–	–	65.1	12.2	–	–
3C Field	48.4	3.7	0.5	2.2	66.4	10.3	1.3	4.0
ADP (Brozek)	48.5	4.0	0.5	2.3	66.3	11.1	1.2	4.5
ADP (Siri)	48.2	3.9	0.3	2.4	66.4	11.0	1.3	4.7
Anthro (F)	48.9	3.8	0.9	3.0	–	–	–	–
MFBIA (InBody)	47.6	3.9	−0.4	3.1	67.4	12.5	2.2	4.5
SFBIA (RJL/Matias)	46.6	4.5	−1.3	2.1	67.0	12.2	1.9	4.2
SFBIA (RJL/Stewart)	–	–	–	–	56.7	11.1	−8.4	5.3
F2FBIA (Tanita)	49.3	5.9	1.4	4.6	67.3	10.6	2.1	5.1
BIS	49.0	5.0	1.0	2.0	66.4	11.8	1.2	6.0
SKF (DL)	53.4	4.1	5.5	2.9	58.0	7.6	−7.1	6.6
SKF (DW)	45.9	3.2	−2.0	2.7	64.9	10.3	−0.2	4.4
SKF (Ev.3)	46.9	3.6	−1.0	2.6	66.9	10.8	1.8	4.0
SKF (Ev.7)	47.8	3.6	−0.2	2.4	67.0	10.3	1.8	4.3
SKF (Forsyth)	48.7	3.8	0.8	4.1	62.2	8.7	−2.9	10.8
SKF (JP3)	48.5	3.6	0.5	2.7	69.1	10.8	4.0	4.0
SKF (JP7)	49.4	3.6	1.4	2.5	68.8	10.5	3.7	4.2
SKF (Katch)	51.6	3.7	3.7	2.6	66.4	9.6	1.3	4.9
SKF (Loftin)	44.4	3.0	−3.5	3.4	–	–	–	–
SKF (Lohman)	51.4	3.7	3.5	2.7	65.7	9.1	0.5	5.3
SKF (Slaughter)	45.8	3.1	−2.1	3.0	–	–	–	–
SKF (Thorland)	51.9	3.6	3.9	2.7	66.8	9.2	1.7	6.0
UWW (Brozek)	47.3	4.1	−0.7	2.2	65.4	11.3	0.3	4.0
UWW (Siri)	47.0	4.2	−0.9	2.3	64.9	11.8	−0.2	4.2

MD, mean difference; SD of MD, SD of mean difference; 3C, 3-compartment model; ADP, air displacement plethysmography; Anthro (F), anthropometric-based equation of Fornetti et al.; MFBIA, multi-frequency bioelectrical impedance analysis; SFBIA, single-frequency bioelectrical impedance analysis; F2FBIA, foot to foot single-frequency bioelectrical impedance analysis; Matias, Matias equation; BIS, bioimpedance spectroscopy; SKF, skinfolds; DL, Devrim-Lanpir equation; DW, Durnin and Womersley equations; Ev.3, Evans 3-site equation; Ev. 7, Evans 7-site equation; Forsyth, Forsyth equation; JP3, Jackson and Pollock 3-site equation; JP7, Jackson and Pollock 7-site equation; Katch, Katch equation; Lohman, Lohman equation; Thorland, Thorland equation; UWW, underwater weighing.

**Figure 1:**
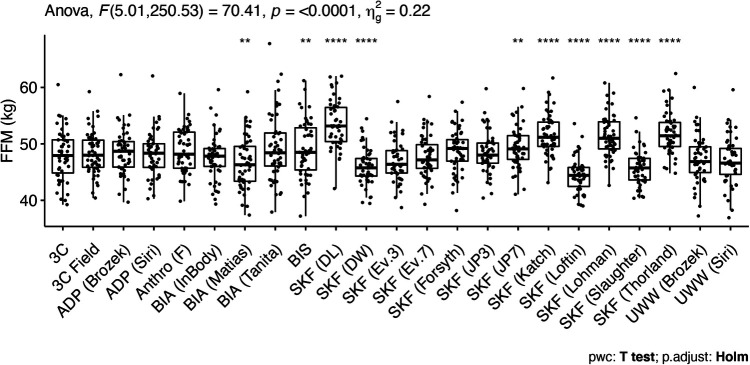
Comparison of fat-free mass values in female athletes. Estimates were compared using one-way analysis of variance with repeated measures. The significant effect of method was followed up with pairwise *t*-tests, using the 3C model as the reference group. The Holm adjustment was performed to correct for multiple comparisons. **Indicates a *p* value between 0.01 and 0.001 and ****indicates a *p* value <0.0001. See footnote on Table 1 for abbreviations.

The ANOVA test indicated significant differences for FFM estimates in females, indicating the following methods differed from the 3C model: SFBIA [RJL/Matias et al. equation (56)], BIS, and the SKF equations of Devrim-Lanpir (26), Durnin and Womersley (38), Jackson and Pollock (7-site) (33), Katch (35), Loftin (42), Lohman (16, 36), Slaughter (43), and Thorland (16, 37) (Figure 1). Equivalence testing indicated that several methods demonstrated equivalence with the reference 3C model based on the ±2 kg equivalence interval. These included: 3C Field, UWW [both Siri (44) and Brozek (45) equations], ADP [both Siri (44) and Brozek (45) equations], BIS, MFBIA (InBody), SFBIA [RJL/Matias equation (56)], the skinfold equations of Forsyth (34), Jackson and Pollock (3-site) (32, 33), Evans (3-site; 7-site) (1), and the anthropometric equation of Fornetti (41).

**Figure 2:**
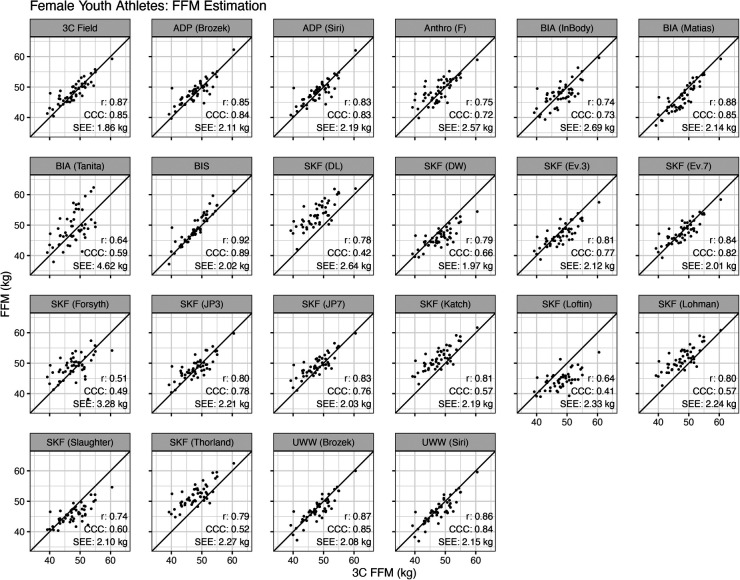
Validity of fat-free mass estimates in female athletes. Each specified method was compared to the reference 3-compartment (3C) model. The Pearson's correlation (*r*), Lin's concordance correlation coefficient (CCC), and standard error of the estimate (SEE) are displayed.

For female athletes, the Pearson's correlations between the reference 3C model and alternate methods ranged from 0.51 to 0.92, the CCC ranged from 0.41 to 0.89, and the SEE ranged from 1.9 to 4.6 kg (Figure 2).

**Figure 3:**
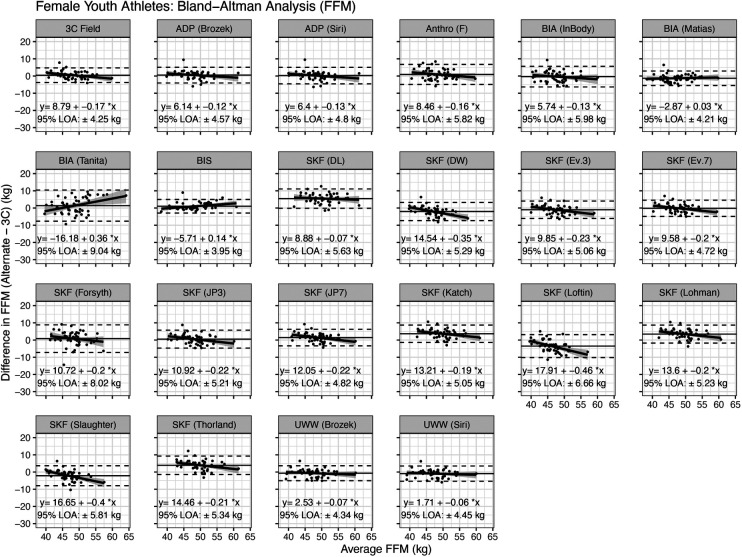
Bland–Altman analysis of fat-free mass estimates in female athletes. Horizontal dashed lines indicate the 95% limits of agreement (i.e., 1.96 times the standard deviation of the difference between methods), and the solid horizontal line indicates the mean difference between methods. The diagonal line indicates the linear relationship between the difference between methods (*y*) and the average of the methods (*x*). A slope significantly different from zero indicates proportional bias. See text for more information.

Bland–Altman analysis indicated that proportional bias was present (i.e., the slope of the linear regression line significantly differed from 0) for the following methods: 3C Field, F2FBIA (Tanita), BIS, and the skinfold equations of Durnin and Womersley (38), Evans 3-site and 7-site equations (1), Jackson and Pollock 3-site and 7-site equations (32, 33), Katch equation (35), Loftin equation (42), Lohman equation (16, 36), Slaughter equation (43), and Thorland equation (16, 37) (Figure 3).

#### 4.1.2. Males

**Figure 4:**
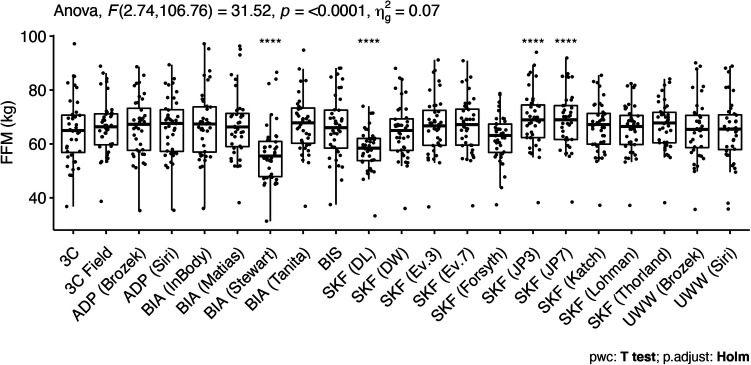
Comparison of fat-free mass values in male athletes. Estimates were compared using one-way analysis of variance with repeated measures. The significant effect of method was followed up with pairwise *t*-tests, using the 3C model as the reference group. The Holm adjustment was performed to correct for multiple comparisons. ****Indicates a *p* value <0.0001. See Figure 1 caption for abbreviations. Stewart, Stewart equation (40).

Significant differences existed for FFM estimates in males (Table 2), indicating that the following methods differed from the 3C model: SFBIA (RJL/Stewart et al.) and the SKF equations of Devrim-Lanpir (26) and Jackson and Pollock (both 3-site and 7-site equations) (32) (Figure 4). Equivalence testing indicated that four methods—UWW [both Siri equation (44) and Brozek equation (45)] and the SKF equations of Lohman (36) and Durnin and Womersley (38)—demonstrated equivalence with the reference 3C model based on the ±2 kg equivalence interval.

**Figure 5:**
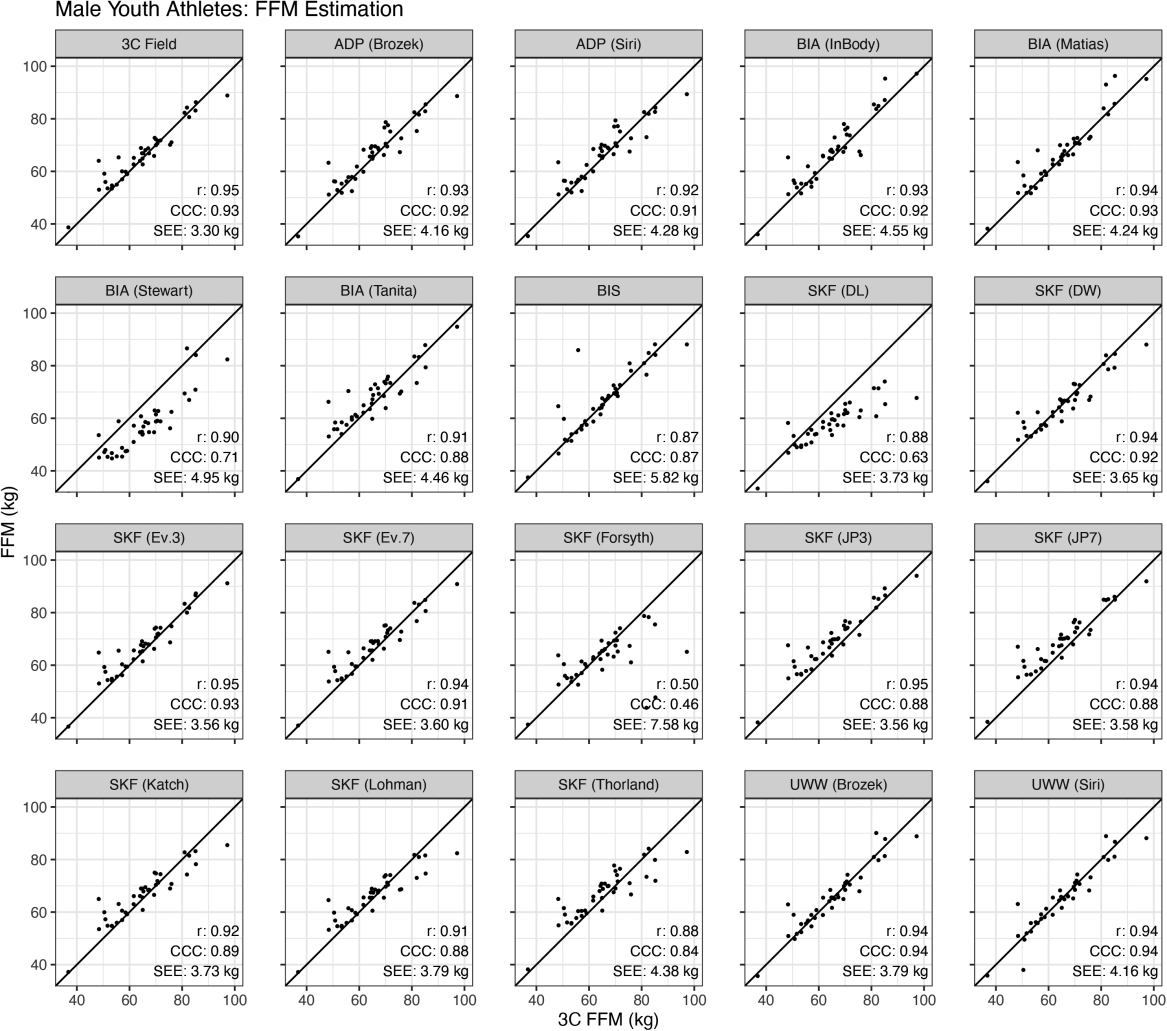
Validity of fat-free mass estimates in male athletes. Each specified method was compared to the reference 3-compartment (3C) model. The Pearson's correlation (*r*), Lin's concordance correlation coefficient (CCC), and standard error of the estimate (SEE) are displayed.

For male athletes, the Pearson's correlations between the reference 3C model and alternate methods ranged from 0.50 to 0.95, the CCC ranged from 0.46 to 0.94, and the SEE ranged from 3.3 to 7.6 kg (Figure 5).

**Figure 6:**
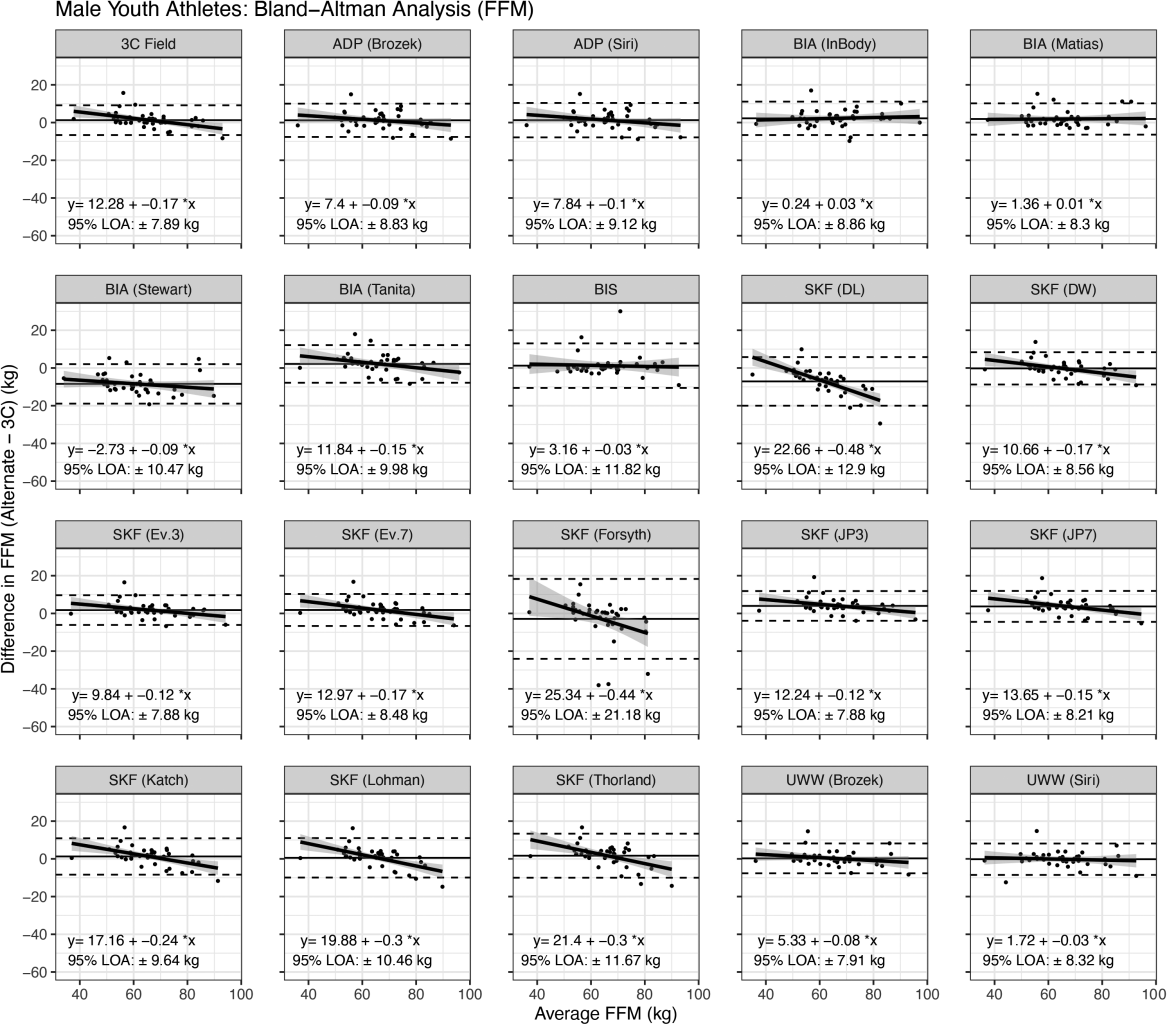
Bland–Altman analysis of fat-free mass estimates in male athletes. Horizontal dashed lines indicate the 95% limits of agreement (i.e., 1.96 times the standard deviation of the difference between methods), and the solid horizontal line indicates the mean difference between methods. The diagonal line indicates the linear relationship between the difference between methods (*y*) and the average of the methods (*x*). A slope significantly different from zero indicates proportional bias. See text for more information.

Bland–Altman analysis indicated that proportional bias was present for the following methods: 3C Field, SFBIA (Tanita), and the skinfold equations of Devrim-Lanpir (26), Durnin and Womersley (38), Evans 3-site and 7-site equations (1), Forsyth (34), Jackson and Pollock 3-site and 7-site equations (32, 33), Katch equation (35), Lohman equation (16, 36), and Thorland equation (16, 37) (Figure 6).

### 4.2. Minimal wrestling weight

As minimal wrestling weight is calculated using measures derived from FFM estimates, the MWW results (see SDC1 for results regarding differences in MWW based upon skinfold prediction equation and impedance analysis device used) are presented in Supplementary Materials only (see SDC2 for Table S5: Minimum Wrestling Weight Estimates for Male and Female Athletes and SDC3 (Figure S7. Comparison of Minimum Wrestling Weight Values in Female Athletes); SDC4 (Figure S8. Validity of Minimum Wrestling Weight Estimates in Female Athletes); SDC5 (Figure S9. Bland–Altman Analysis of Minimum Wrestling Weight Estimates in Female Athletes.); SDC6 (Figure S10. Comparison of Minimum Wrestling Weight Values in Male Athletes.); SDC7 (Figure S11. Validity of Minimum Wrestling Weight Estimates in Male Athletes); and SDC8 (Figure S12. Bland–Altman Analysis of Minimum Wrestling Weight Estimates in Male Athletes.), in an effort to avoid redundancy.

## 5. Discussion

The current study had two primary aims: (A) to determine the most accurate skinfold prediction equations for young male and female athletes using a three-compartment model of body composition assessment; and (B) to examine the utility of alternative modes of body composition assessment compared to criterion measures. This is the first study to examine the validity of skinfold prediction equations in young male and female athletes. The main findings indicate multiple discrepancies in FFM estimates for female and male athletes when compared to the 3C model. In females, The Evans 3 and 7-site, Forsyth, and Jackson and Pollock (3-site) SKF prediction equations performed best, while the Evans 3-site equation appeared to perform best when determining FFM in male athletes. Additionally, the field 3C model can provide a suitable alternative measure of FFM for both male and female athletes when laboratory-grade criterion measures are not available.

In females, the SKF prediction equations of Devrim-Lanpir (26), Durnin and Womersley (38), Jackson and Pollock (7-site) (33), Katch (35), Loftin (42), Lohman (16, 36), Slaughter (43), and Thorland (16, 37) differed from the 3C model (Figure 1). However, equivalence testing indicated that the equations of Forsyth (34), Jackson and Pollock (3-site) (32, 33), Evans (3-site and 7-site) (1), and the anthropometric equation of Fornetti (41) demonstrated equivalence with the reference 3C model based on the ±2 kg equivalence interval. In the context of wrestling and MWW determination, this suggests the estimates of FFM and subsequently MWW are likely to fall within the limits of each weight class division (often in 5.4 kg increments) utilizing these SKF equations. However, this could impact wrestlers who are on the threshold of a certain MWW and weight class. There was evidence of proportional bias for the skinfold equations of Durnin and Womersley (38), Evans 3-site and 7-site equations (1), Jackson and Pollock 3-site and 7-site (32, 33), Katch (35), Loftin (42), Lohman (16, 36), Slaughter (43), and Thorland (16, 37) (Figure 3). The Evans 3 and 7-site, Forsyth, and Jackson and Pollock (3-site) SKF prediction equations performed best with mean differences (SEE) values of −1.0 (2.12) kg, −0.2 (2.01) kg, 0.8 (3.28), and 0.5 (2.21), respectively. When taking concordance correlation coefficient (CCC) values into consideration, the Evans 7-site SKF equation performed best (CCC = 0.82). Collectively, these findings indicate the Evans 7-site equation appears to perform best among SKF prediction equations for female athletes when determining FFM. If a 3-site method is preferred for ease of use, the J&P and Evans 3-site equations produced the next highest CCC values of 0.78 and 0.77, respectively, and with mean differences (SEE) of 0.5 (2.21) kg and −1.0 (2.12) kg.

The current MWW certification process for girls' high school wrestling in Wisconsin requires the use of the Slaughter SKF equation (43), which resulted in a mean difference (SEE) of −2.1 kg (2.1) kg, a CCC value of 0.60 when compared to criterion measures in the current study. Additionally, there was a proportional bias towards a greater underestimation of FFM for athletes with higher FFM values, which could subsequently result in a higher estimate of BF% and lower MWW. This could potentially allow a female wrestler to compete in a lower weight class than what would be allowed if FFM was assessed more accurately. When evaluating the number of female athletes that would be mis-categorized when determining MWW, the current method would allow 31/51 (60.8%) of the current female athletes to compete in a weight class that would be different from the criterion-derived MWW and resultant weight class.

Among the remaining body composition assessment modalities, no differences were observed between 3C Field, ADP [both Siri (44) and Brozek (45) equations], nor the UWW (Brozek and Siri equations) compared to the criterion 3C model when determining FFM for females. The 3C Field resulted in a mean difference (SEE) of 0.5 (1.86) kg and the highest CCC (0.85) among all methods. However, there was proportional bias for the 3C Field, indicating that the model tended to overestimate FFM in those with low FFM levels but underestimate FFM in those with higher FFM. However, it should also be noted that the performance of the Field 3C model is dependent upon the field methods used to estimate *D_b_* and TBW, so alternate versions of this model may produce dissimilar results. The 3C Field, UWW [both Siri (44) and Brozek (45) equations], ADP [both Siri (44) and Brozek (45) equations] all demonstrated equivalence with the reference 3C model. For impedance analysis, the SFBIA [RJL/Matias et al. equation (56)], and BIS differed from the 3C model; however, equivalence testing indicated the BIS, MFBIA (InBody), and SFBIA [RJL/Matias equation (56)] all demonstrated equivalence with the reference 3C model using the 2.5 kg threshold when determining FFM for female athletes. However, there was also proportional bias for the F2FBIA (Tanita), and BIS, which again indicates a tendency to overestimate measures of FFM in those with higher FFM.

In male athletes, the FFM values derived from the SKF equations of Devrim-Lanpir (26) and Jackson and Pollock (both 3-site and 7-site equations) (32) differed from the 3C model (Figure 4) while proportional bias was present for the Devrim-Lanpir (26), Durnin and Womersley (38), Evans 3-site and 7-site (1), Forsyth (34), Jackson and Pollock 3-site and 7-site (32, 33), Katch (35), Lohman (16, 36), and Thorland equations (16, 37) (Figure 6). The equations of Lohman (36) and Durnin and Womersley (38) demonstrated equivalence with the reference 3C model based on the ±2 kg equivalence interval and the Evans 3-site equation appeared to perform best with a mean difference (SEE) of 1.8 (3.56) kg and a CCC of 0.93. The current MWW certification process for high school boys wrestling in Wisconsin utilizes the Lohman equation, which comparatively, resulted in a mean difference (SEE) of 0.5 (3.79) kg and a CCC of 0.88. Additionally, the Lohman equation tended to overestimate FFM, which would subsequently underestimate BF% and result in a higher MWW than would occur with a more accurate measure of FFM. When evaluating the number of male athletes that would be mis-categorized when determining MWW, the current method would allow 29/41 (72.5%) of the current male athletes to compete in a weight class that would be different from the criterion-derived MWW and resultant weight class.

When estimating FFM for the male athletes using the alternative methods, equivalence testing indicated that UWW [both Siri equation (44) and Brozek equation (45)] demonstrated equivalence with the reference 3C model based on the ±2 kg equivalence interval. The Field 3C model resulted in a mean difference (SEE) of 1.3 (3.30) kg and a CCC of 0.93, serving as a suitable alternative for a laboratory-grade 3C model. However, proportional bias was present for the 3C Field, with a tendency to overestimate FFM in those with lower FFM but underestimate FFM in those with higher FFM. For impedance analysis, only the SFBIA [RJL/Stewart et al. (40)] differed from the 3C model suggesting that the impedance devices used in the current study appear to be more accurate for the determination measures of FFM in male athletes compared to females, with correlation coefficients ranging from 0.87 to 0.94, CCC values of 0.88–0.93 and SEE ranging from 4.24 to 5.82 kg. Proportional bias was present for the F2FBIA (Tanita) indicating greater underestimation of FFM values in those with higher FFM. FFM was underestimated for most males by Tanita and became more pronounced as FFM increased as indicated by the negative slope of the Bland–Altman line (Figure 6).

Previous research in college-age men (25) reported discrepancies in MWW values with SEEs of 3.2, 3.4, and 2.4 kg for ADP, DXA, and ultrasound, respectively when compared with SKF. Further, compared to DXA and ultrasound measures, reliance on SKF-derived MWW would allow wrestlers to certify at a lower weight class 64% and 33% of the time, respectively, which is in opposition of what weight certification programs are designed to accomplish (57). When comparing the current NCAA approved methods (SKF and ADP) for MWW determination, approximately 50% of the male subjects would have certified at a different weight class depending on the method used (57). These findings, in addition to the ones from the current study indicate the potential variability in FFM, and subsequently MWW, through the use of different methods of BF% assessment.

When evaluating more practical methods of BF% assessment in high school athletes, no differences were reported in high school wrestlers between a similar MFBIA unit as the one used in the current study and the UWW criterion methods with a SEE for FFM of 2.73 kg and correlation coefficient of *r* = 0.96 (3). Clark et al. (58) reported similar mean values (72.2 ± 9.7 vs. 72.2 ± 10.3 kg), and a high correlation (*r* = 0.94) between BIA and a criterion 4C model. However, the authors (58) reported large individual differences and systematic bias across the range of MWW values. Additionally, the BIA was able to predict MWW within 3.5 kg 68% of the time and within 7.0 kg 95% of the time. Others reported no differences in MWW from UWW (70.5 ± 7.3 kg, *P *= 0.57), SKF (70.5 ± 7.2 kg, *P *= 0.29), BIA (70.6 ± 7.6 kg, *P *= 0.39), DXA (70.3 ± 7.5, *P *= 0.97), and the 4C criterion (70.3 ± 7.4 kg) in male collegiate wrestlers (59). The UWW and SKF exhibited the highest degree of precision (lowest SEE) with SEE values of 1.31 and 1.72 kg, respectively, compared to the 4C criterion. Therefore, it is possible that with more advanced models used for criterion measures, there is more agreement between alternative BF% assessment techniques thereby resulting in a more appropriate minimal wrestling weight. In most high school settings, SKF is likely the modality of choice because of its low cost and ease of use. When limiting the scope to SKF and *D_b_* prediction equations used to estimate BF%, previous research (26) in Olympic wrestlers (age: 12–20 years) found that the Stewart and Hannan equation (40) for male wrestlers and the Durnin and Womersley equation (38) for female wrestlers were the most accurate, least biased, and positively correlated with the criterion measure using ADP. Conversely, Clark et al. (14) found that Lohman1 (*r* = 0.961; TE = 2.2 kg), Lohman2 (*r* = 0.920; TE = 2.6 kg) and Katch (*r *= 0.969; TE = 1.7 kg) equations were the most accurate with smaller mean differences, smaller total error, and higher correlation compared to three other SKF prediction equations when estimating MWW in male high school wrestlers compared to UWW. A similar study in collegiate male wrestlers found the Lohman equation to be a valid measure of BF% (SEE = 2.32 kg; TE = 2.49 kg), which is the currently accepted method for NCAA collegiate wrestling. In high school wrestlers, the Lohman SKF equation was found to be a valid measure of FFM with a SEE of 2.66 kg and correlation coefficient of *r* = 0.97. It is possible the physiques and anthropometric characteristics of youth athletes may have changed over the past decades, thereby influencing the ability of certain SKF prediction equations or alternative modalities to accurately estimate BF%. Furthermore, impedance devices may have limitations with athletic populations, as previous research has indicated that generalized impedance-based equations underestimate body fluids in athletes, potentially influencing measures of FFM. Future investigations in a large, mixed-sex group could provide new equations (SKF and impedance) for estimating FFM in youth athletes.

## 6. Conclusions

Results from the current study indicate the Evans 7-site and 3-site SKF equations performed best for female and male athletes, respectively. The current MWW certification process for girls' high school wrestling in Wisconsin does not appear to utilize the best SKF prediction equation available for this population. Additionally, there was a proportional bias towards an underestimation of FFM, which could subsequently result in a higher estimate of BF% and lower MWW. This could permit a female wrestler to compete in a lower weight class than what would be allowed if FFM was assessed more accurately. For male wrestlers in Wisconsin, the Lohman equation is currently used, which provided an adequate estimate of FFM yet was not the best performing SKF prediction equation. Additionally, the Lohman equation tended to overestimate FFM, which would subsequently underestimate BF% and result in a higher MWW than would occur with a more accurate measure of FFM. The field 3C model can provide a suitable alternative measure of FFM for both male and female athletes when laboratory-grade criterion measures are not available. Athletic organizations, specifically wrestling programs, should explore the feasibility of implementing field-based 3C models of SKF in conjunction with BIA units to improve upon the current assessment of BF%.

## Data Availability

The datasets associated with the current manuscript are not readily available as additional analysis is pending. Partial data may be available upon request.
