# Supplementary material for: Validation of skinfold equations and alternative methods for the determination of fat-free mass in young athletes
Source: Front Sports Act Living. 2023 Aug 11;5:1240252. doi: 10.3389/fspor.2023.1240252 (PMC10453806; doi:10.3389/fspor.2023.1240252)
Supplement: Supplementary file 1 [file DataSheet1.zip › Supplementary Results.DOCX]

**Minimum Wrestling Weight**

*Females*

MWW estimates for both sexes are displayed in Table 2. The ANOVA test indicated significant differences for estimates in females, with follow up testing indicating that the following methods significantly differed from the 3C model: BIA (Matias et al. equation[1]), BIS, and the SKF equations of Devrim-Lanpir[2], Durnin and Womersley[3], Jackson and Pollock (7-site)[4], Katch[5, 6], Loftin[7], Lohman[5, 8], Slaughter[9, 10], and Thorland[5, 11] (Figure 7). Equivalence testing indicated that several methods demonstrated equivalence with the reference 3C model based on the ±2 kg equivalence interval. These included: 3C Field, UWW (both Siri[12] and Brozek[13] equations), ADP (both Siri[12] and Brozek[13] equations), BIS, BIA (InBody), the skinfold equations of Jackson and Pollock (3-site)[4, 14], Evans (3-site and 7-site)[15], and the anthropometric equation of Fornetti[16].

For female athletes, the Pearson’s correlations between the reference 3C model and alternate methods ranged from 0.51 to 0.92, the CCC ranged from 0.41 to 0.90, and the SEE ranged from 2.1 to 5.2 kg (Figure 8). Bland-Altman analysis indicated that proportional bias was present (i.e., the slope of the linear regression line significantly differed from 0) for the following methods: 3C Field, BIA (Tanita), BIS, and the skinfold equations of Durnin and Womersley[3], Evans 3-site and 7-site equations[15], Jackson and Pollock 3-site and 7-site equations[4, 14], Katch equation[6], Loftin equation[7], Lohman equation[5, 8], Slaughter equation[9, 10], and Thorland equation [5, 11] (Figure 9).

*Males*

The ANOVA test indicated significant differences for estimates in males, with follow up testing indicating that the following methods significantly differed from the 3C model: BIA (Stewart et al.) and the SKF equations of Devrim-Lanpir[2] and Jackson and Pollock (both 3-site and 7-site equations)**[14]** (Figure 10). Equivalence testing indicated that four methods – UWW (both Siri equation[12] and Brozek equation[13]), BIS, and the SKF equation of Durnin and Womersley[3] – demonstrated equivalence with the reference 3C model based on the ±2 kg equivalence interval.

For male athletes, the Pearson’s correlations between the reference 3C model and alternate methods ranged from 0.50 to 0.95, the CCC ranged from 0.46 to 0.95, and the SEE ranged from 3.6 to 8.1 kg (Figure 11). Bland-Altman analysis indicated that proportional bias was present for the following methods: 3C Field, BIA (Tanita), and the skinfold equations of Devrim-Lanpir[2], Durnin and Womersley[3], Evans 3-site and 7-site equations[15], Forsyth[17], Jackson and Pollock 3-site and 7-site equations[4, 14], Katch equation[6], Lohman equation[5, 8], and Thorland equation [5, 11] (Figure 12).

**References**

1. Matias, C.N., et al., *Fat-free Mass Bioelectrical Impedance Analysis Predictive Equation for Athletes using a 4-Compartment Model.* Int J Sports Med, 2020.

2. Devrim-Lanpir, A., et al., *Which Body Density Equations Calculate Body Fat Percentage Better in Olympic Wrestlers?-Comparison Study with Air Displacement Plethysmography.* Life (Basel), 2021. **11**(7).

3. Durnin, J.V. and J. Womersley, *Body fat assessed from total body density and its estimation from skinfold thickness: measurements on 481 men and women aged from 16 to 72 years.* Br J Nutr, 1974. **32**(1): p. 77-97.

4. Jackson, A.S., M.L. Pollock, and A. Ward, *Generalized equations for predicting body density of women.* Med Sci Sports Exerc, 1980. **12**(3): p. 175-81.

5. Thorland, W.G., et al., *Midwest wrestling study: prediction of minimal weight for high school wrestlers.* Med Sci Sports Exerc, 1991. **23**(9): p. 1102-10.

6. Katch, F.I. and W.D. McArdle, *Prediction of Body Density from Simple Anthropometric Measurements in College-Age Men and Women.* Human Biology, 1973. **45**(3): p. 445-455.

7. Loftin, M., et al., *Comparison of the validity of anthropometric and bioelectric impedance equations to assess body composition in adolescent girls.* Int J Body Compos Res, 2007. **5**(1): p. 1-8.

8. Lohman, T.G., *Skinfolds and body density and their relation to body fatness: a review.* Hum Biol, 1981. **53**(2): p. 181-225.

9. Truesdale, K.P., et al., *Comparison of Eight Equations That Predict Percent Body Fat Using Skinfolds in American Youth.* Child Obes, 2016. **12**(4): p. 314-23.

10. Slaughter, M.H., et al., *Skinfold equations for estimation of body fatness in children and youth.* Hum Biol, 1988. **60**(5): p. 709-23.

11. Thorland, W.G., et al., *Estimation of body density in adolescent athletes.* Hum Biol, 1984. **56**(3): p. 439-48.

12. Siri, W.E., *The gross composition of the body.* Adv Biol Med Phys., 1956. **4**: p. 239-80.

13. Brozek, J., et al., *Densitometric analysis of body composition: revision of some quantitative assumptions.* Ann N Y Acad Sci., 1963. **110**: p. 113-40.

14. Jackson, A.S. and M.L. Pollock, *Generalized equations for predicting body density of men.* Br J Nutr, 1978. **40**(3): p. 497-504.

15. Evans, E.M., et al., *Skinfold prediction equation for athletes developed using a four-component model.* Med Sci Sports Exerc, 2005. **37**(11): p. 2006-11.

16. Fornetti, W.C., et al., *Reliability and validity of body composition measures in female athletes.* J Appl Physiol (1985), 1999. **87**(3): p. 1114-22.

17. Forsyth, H.L. and W.E. Sinning, *The anthropometric estimation of body density and lean body weight of male athletes.* Med Sci Sports, 1973. **5**(3): p. 174-80.

18. Stewart, A.D. and W.J. Hannan, *Prediction of fat and fat-free mass in male athletes using dual X-ray absorptiometry as the reference method.* J Sports Sci, 2000. **18**(4): p. 263-74.
