# Supplementary material for: Validation of skinfold equations and alternative methods for the determination of fat-free mass in young athletes
Source: Front Sports Act Living. 2023 Aug 11;5:1240252. doi: 10.3389/fspor.2023.1240252 (PMC10453806; doi:10.3389/fspor.2023.1240252)
Supplement: Supplementary file 1 [file DataSheet1.zip › Supplementary Table.DOCX]

**Minimum Wrestling Weight**

**Table 5. Minimum Wrestling Weight Estimates**

|  | **Females (n=51)** | | | | **Males (n=40)** | | | |
| --- | --- | --- | --- | --- | --- | --- | --- | --- |
| **Method** | **Mean** | **SD** | **MD** | **SD of MD** | **Mean** | **SD** | **MD** | **SD of MD** |
| 3C | 54.5 | 5.0 | - | - | 70.0 | 13.1 | - | - |
| 3C Field | 55.0 | 4.3 | 0.5 | 2.5 | 71.4 | 11.1 | 1.4 | 4.3 |
| ADP (Brozek) | 55.1 | 4.5 | 0.6 | 2.7 | 71.3 | 12.0 | 1.3 | 4.8 |
| ADP (Siri) | 54.9 | 4.6 | 0.4 | 2.8 | 71.4 | 11.8 | 1.3 | 5.0 |
| Anthro (F) | 55.5 | 4.4 | 1.1 | 3.4 | - | - | - | - |
| BIA (InBody) | 54.1 | 4.5 | -0.4 | 3.5 | 72.4 | 13.5 | 2.4 | 4.9 |
| BIA (Matias) | 53.0 | 5.2 | -1.5 | 2.4 | 72.1 | 13.2 | 2.0 | 4.6 |
| BIA (Stewart) | - | - | - | - | 61.0 | 12.0 | -9.1 | 5.7 |
| BIA (Tanita) | 56.1 | 6.7 | 1.6 | 5.2 | 72.3 | 11.4 | 2.3 | 5.5 |
| BIS | 55.6 | 5.6 | 1.1 | 2.2 | 70.5 | 12.3 | 0.5 | 4.1 |
| SKF (DL) | 60.7 | 4.7 | 6.2 | 3.3 | 62.4 | 8.2 | -7.7 | 7.1 |
| SKF (DW) | 52.2 | 3.6 | -2.3 | 3.1 | 69.8 | 11.1 | -0.2 | 4.7 |
| SKF (Ev.3) | 53.3 | 4.1 | -1.1 | 2.9 | 71.9 | 11.6 | 1.9 | 4.3 |
| SKF (Ev.7) | 54.3 | 4.1 | -0.2 | 2.7 | 72.0 | 11.1 | 2.0 | 4.7 |
| SKF (Forsyth) | 55.4 | 4.3 | 0.9 | 4.7 | 66.9 | 9.3 | -3.1 | 11.6 |
| SKF (JP3) | 55.1 | 4.1 | 0.6 | 3.0 | 74.3 | 11.6 | 4.3 | 4.3 |
| SKF (JP7) | 56.1 | 4.1 | 1.6 | 2.8 | 74.0 | 11.3 | 4.0 | 4.5 |
| SKF (Katch) | 58.6 | 4.2 | 4.2 | 2.9 | 71.4 | 10.3 | 1.4 | 5.3 |
| SKF (Loftin) | 50.5 | 3.4 | -4.0 | 3.9 | - | - | - | - |
| SKF (Lohman) | 58.4 | 4.2 | 3.9 | 3.0 | 70.6 | 9.8 | 0.6 | 5.7 |
| SKF (Slaughter) | 52.0 | 3.5 | -2.4 | 3.4 | - | - | - | - |
| SKF (Thorland) | 58.9 | 4.1 | 4.5 | 3.1 | 71.8 | 9.8 | 1.8 | 6.4 |
| UWW (Brozek) | 53.7 | 4.7 | -0.8 | 2.5 | 70.3 | 12.1 | 0.3 | 4.3 |
| UWW (Siri) | 53.4 | 4.7 | -1.0 | 2.6 | 69.8 | 12.7 | -0.2 | 4.6 |

*Abbreviations*: MD – mean difference, SD of MD – SD of mean difference, 3C – 3-compartment model, ADP – air displacement plethysmography, Anthro (F) – anthropometric-based equation of Fornetti et al.^1^, BIA – bioelectrical impedance analysis, Matias – Matias equation^2^, BIS – bioimpedance spectroscopy, SKF – skinfolds, DL – Devrim-Lanpir equation^3^, DW – Durnin and Womersley equations^4^, Ev.3 – Evans 3-site equation^5^, Ev. 7 – Evans 7-site equation^5^, Forsyth – Forsyth equation^6^, JP3 – Jackson and Pollock 3-site equation^7,8^, JP7 – Jackson and Pollock 7-site equation^7,8^, Katch – Katch equation^9^, Lohman – Lohman equation^10^, Thorland – Thorland equation^11^, UWW – underwater weighing.

**REFERENCES**

1. Fornetti WC, Pivarnik JM, Foley JM, Fiechtner JJ. Reliability and validity of body composition measures in female athletes. *J Appl Physiol (1985).* 1999;87(3):1114-1122.

2. Matias CN, Campa F, Santos DA, Lukaski H, Sardinha LB, Silva AM. Fat-free Mass Bioelectrical Impedance Analysis Predictive Equation for Athletes using a 4-Compartment Model. *Int J Sports Med.* 2020.

3. Devrim-Lanpir A, Badem EA, Işık H, et al. Which Body Density Equations Calculate Body Fat Percentage Better in Olympic Wrestlers?-Comparison Study with Air Displacement Plethysmography. *Life (Basel).* 2021;11(7).

4. Durnin JV, Womersley J. Body fat assessed from total body density and its estimation from skinfold thickness: measurements on 481 men and women aged from 16 to 72 years. *Br J Nutr.* 1974;32(1):77-97.

5. Evans EM, Rowe DA, Misic MM, Prior BM, Arngrímsson SA. Skinfold prediction equation for athletes developed using a four-component model. *Med Sci Sports Exerc.* 2005;37(11):2006-2011.

6. Forsyth HL, Sinning WE. The anthropometric estimation of body density and lean body weight of male athletes. *Med Sci Sports.* 1973;5(3):174-180.

7. Jackson AS, Pollock ML. Generalized equations for predicting body density of men. *Br J Nutr.* 1978;40(3):497-504.

8. Jackson AS, Pollock ML, Ward A. Generalized equations for predicting body density of women. *Med Sci Sports Exerc.* 1980;12(3):175-181.

9. Katch FI, McArdle WD. Prediction of Body Density from Simple Anthropometric Measurements in College-Age Men and Women. *Human Biology.* 1973;45(3):445-455.

10. Lohman TG. Skinfolds and body density and their relation to body fatness: a review. *Hum Biol.* 1981;53(2):181-225.

11. Thorland WG, Johnson GO, Tharp GD, Housh TJ, Cisar CJ. Estimation of body density in adolescent athletes. *Hum Biol.* 1984;56(3):439-448.
