# Supplementary figures and images for: Validation of skinfold equations and alternative methods for the determination of fat-free mass in young athletes
Source: Front Sports Act Living. 2023 Aug 11;5:1240252. doi: 10.3389/fspor.2023.1240252 (PMC10453806; doi:10.3389/fspor.2023.1240252)

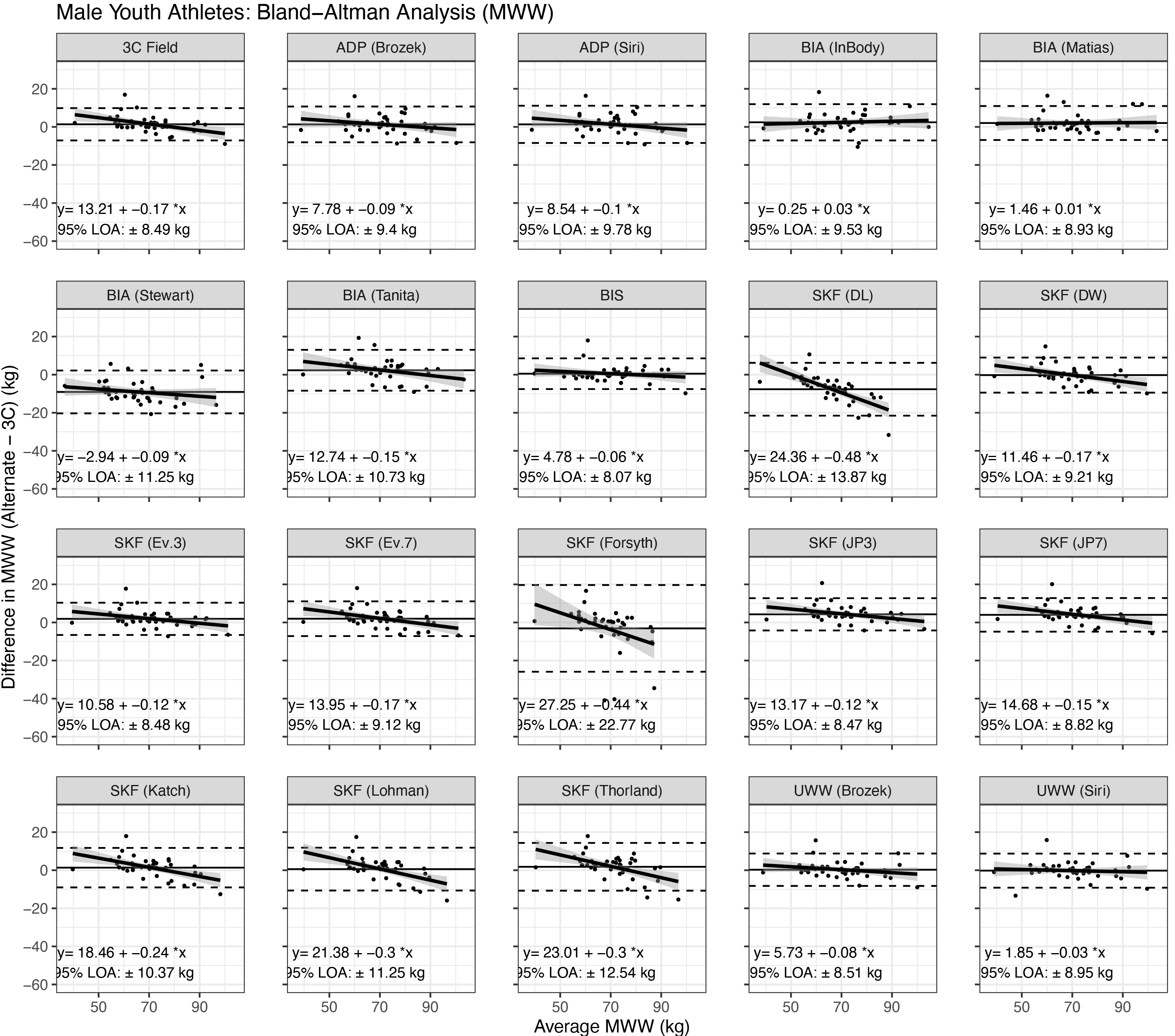

Supplement: Supplementary file 1 [file DataSheet1.zip › Image 1.TIF]

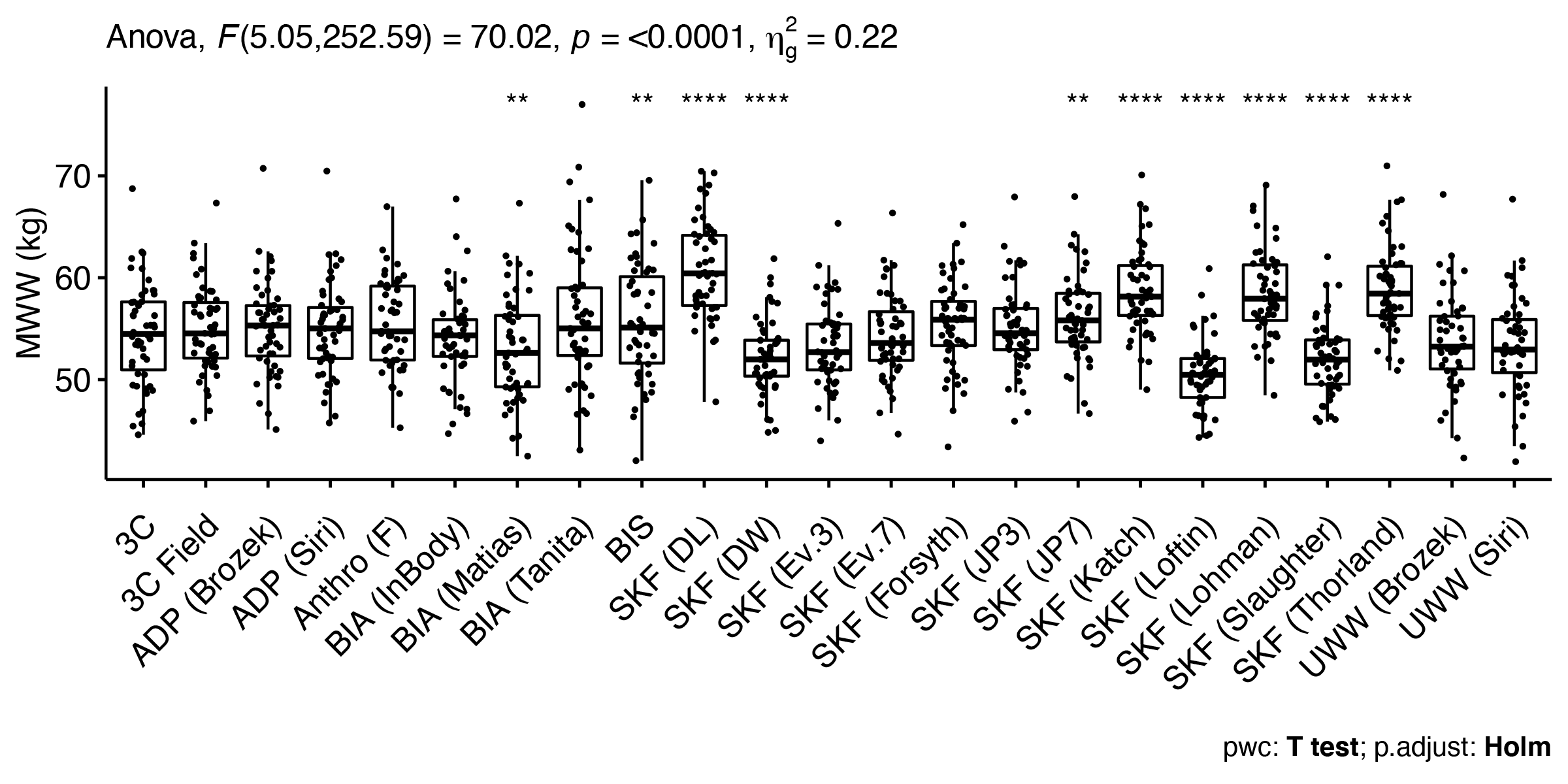

Supplement: Supplementary file 1 [file DataSheet1.zip › Image 2.TIF]

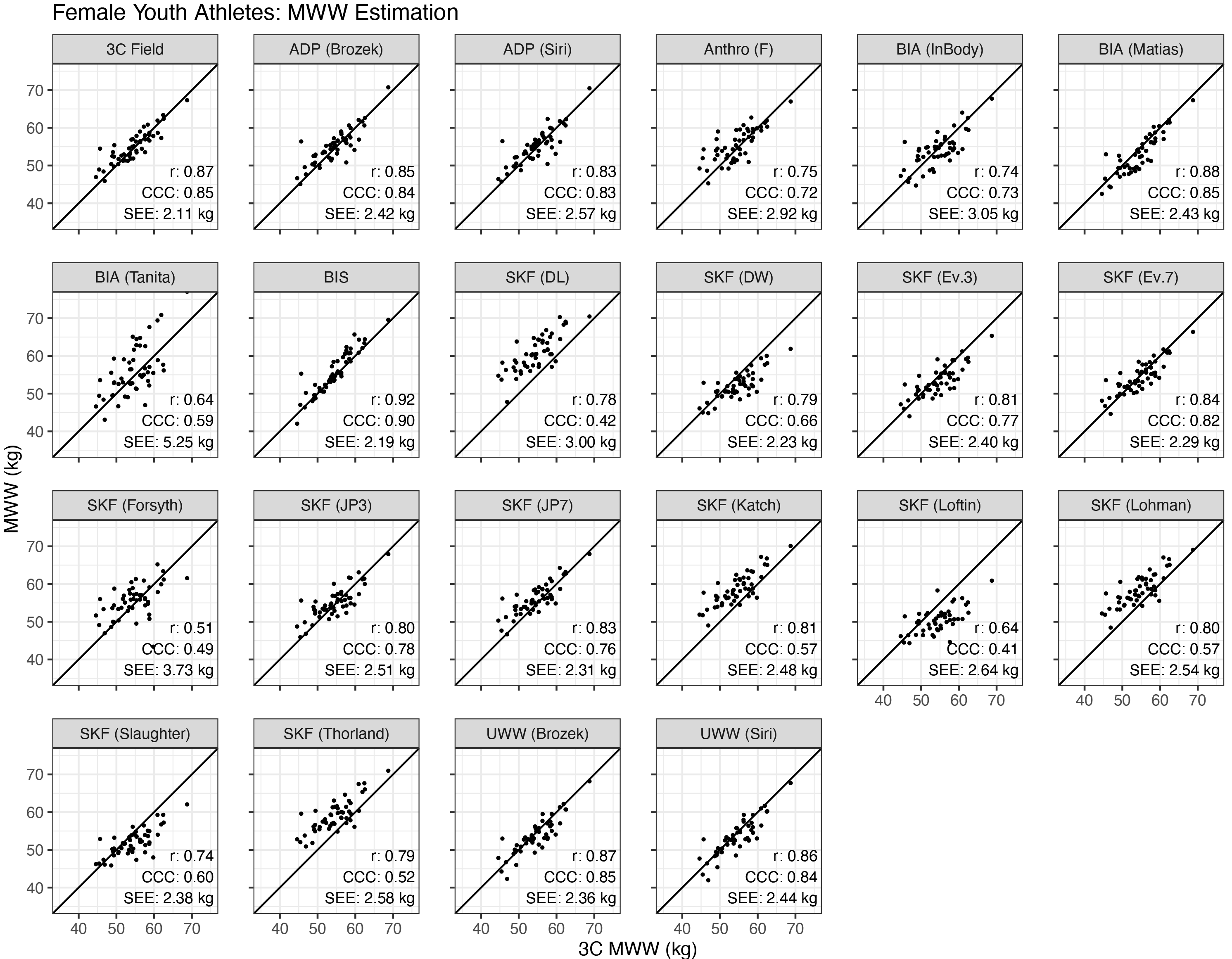

Supplement: Supplementary file 1 [file DataSheet1.zip › Image 3.TIF]

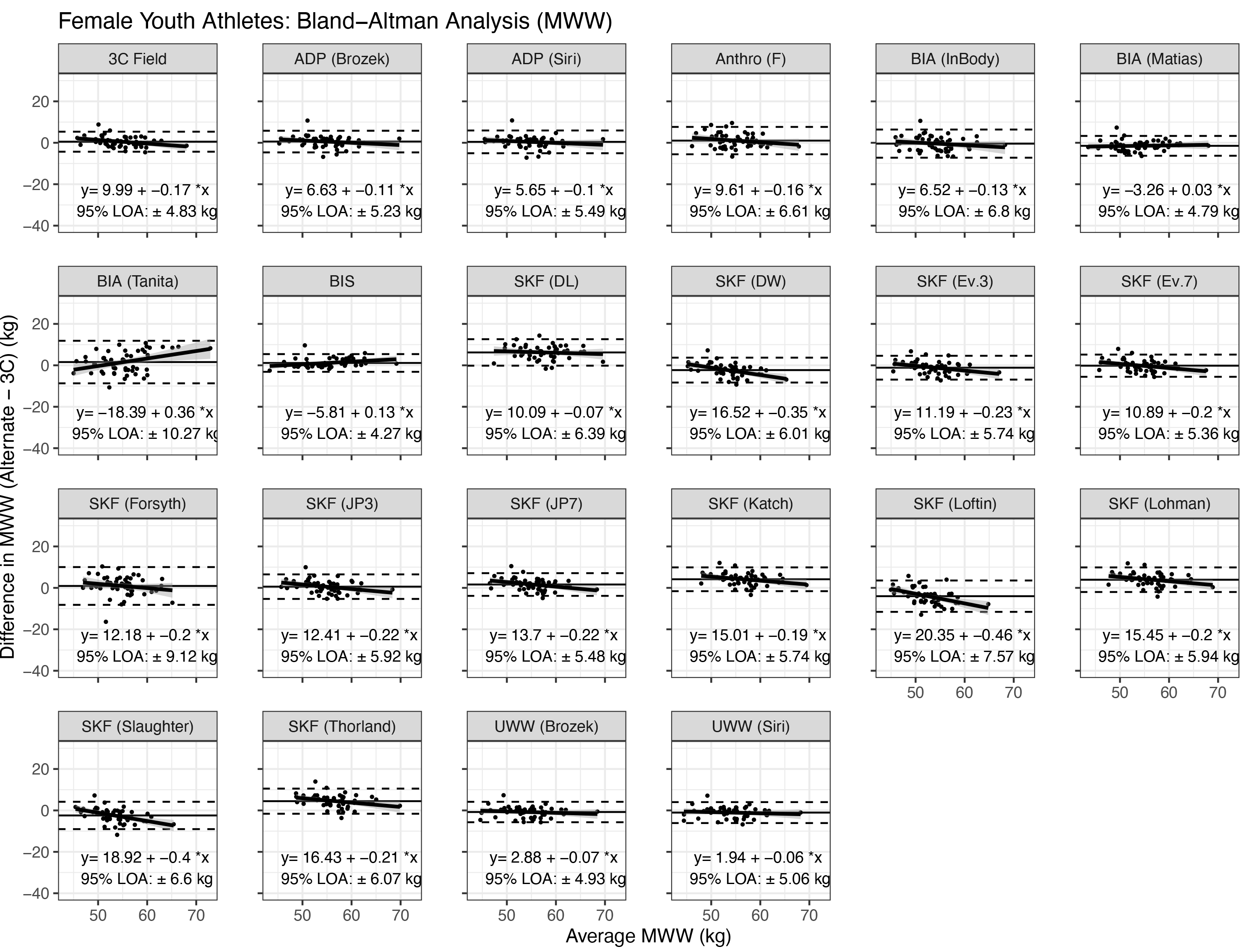

Supplement: Supplementary file 1 [file DataSheet1.zip › Image 4.TIF]

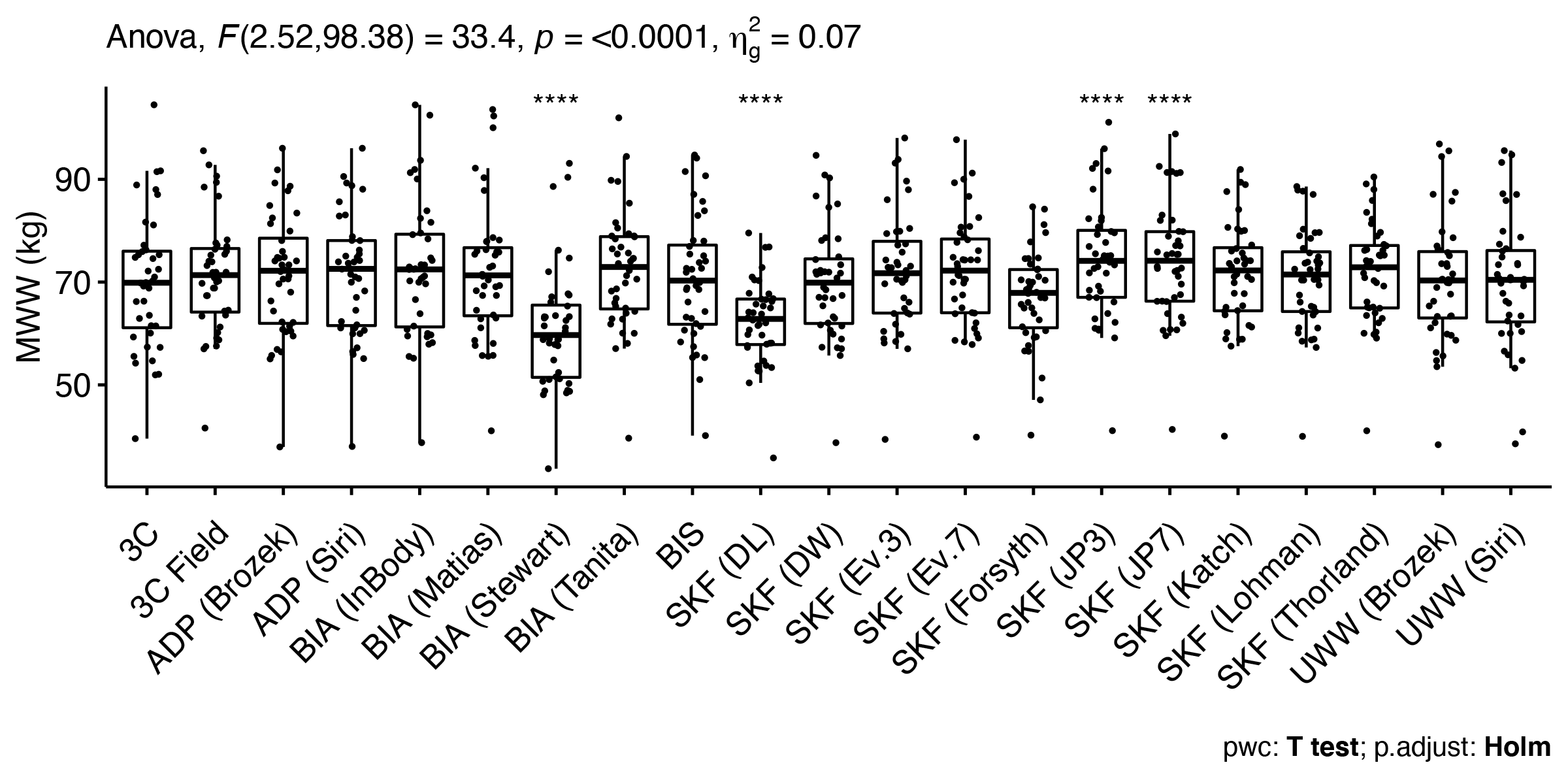

Supplement: Supplementary file 1 [file DataSheet1.zip › Image 5.TIF]

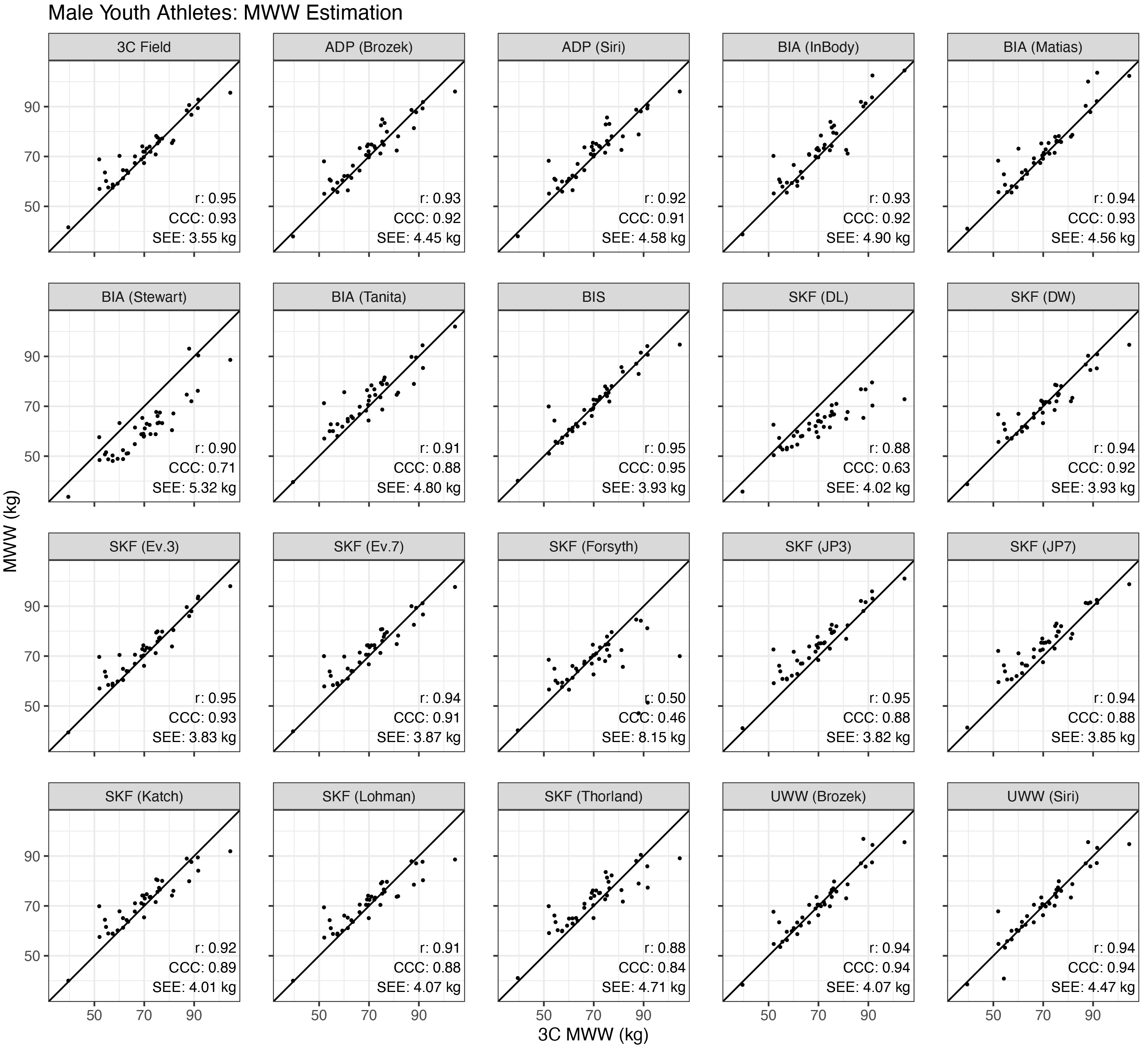

Supplement: Supplementary file 1 [file DataSheet1.zip › Image 6.TIF]
